# NMR in target driven drug discovery: why not?

**DOI:** 10.1007/s10858-020-00343-9

**Published:** 2020-09-08

**Authors:** Sébastien Keiffer, Marta G. Carneiro, Johan Hollander, Masakazu Kobayashi, Denys Pogoryelev, Eiso AB, Stephan Theisgen, Gerhard Müller, Gregg Siegal

**Affiliations:** 1 ZoBio, JH Oortweg 19, 2333CH Leiden, Netherlands; 2 Gotham GmbH, Am Klopferspitz 19a, 82152 Martinsried, Germany; 3 Amsterdam Institute of Molecular and Life Sciences, Free University Amsterdam, De Boelelaan 1108, 1081 HZ Amsterdam, The Netherlands

**Keywords:** Small molecule drug discovery, Biophysics, Structure based drug design, Fragment based drug discovery

## Abstract

No matter the source of compounds, drug discovery campaigns focused directly on the target are entirely dependent on a consistent stream of reliable data that reports on how a putative ligand interacts with the protein of interest. The data will derive from many sources including enzyme assays and many types of biophysical binding assays such as TR-FRET, SPR, thermophoresis and many others. Each method has its strengths and weaknesses, but none is as information rich and broadly applicable as NMR. Here we provide a number of examples of the utility of NMR for enabling and providing ongoing support for the early pre-clinical phase of small molecule drug discovery efforts. The examples have been selected for their usefulness in a commercial setting, with full understanding of the need for speed, cost-effectiveness and ease of implementation.

## Introduction

Some twelve years ago one of us contributed to a far-reaching perspective describing a number of powerful ways in which NMR can contribute to the early stages of developing new small molecule drugs (Pellecchia 2008). In the intervening years, NMR has become an ever more deeply embedded part of our drug discovery process (Glas, et al. 2019; Carneiro 2017; Pritisanac 2017; Chaikuad 2016). However, the use of NMR outside of synthetic chemistry remains confined to a subset of large pharmaceutical companies.

In this short perspective, we provide a number of examples in which relatively simple NMR experiments routinely provide us with data critical to the setup of new programs, interpretation of biophysical and biochemical data and elucidation of structural information describing the protein-small molecule interaction. Many of these examples will seem obvious to the reader, and yet we observe over and over again that the fundamental importance of this type of information is often neglected. These experiments have been selected based on realistic criteria, that is they should: minimize protein consumption, be robust and maximize throughput. The ligand observed experiments do not require particularly high field strength, but an automated sample changer is a must and a cryoprobe is highly desirable. Lastly, the data should be readily interpretable and therefore actionable. Partly, this depends on the skill of the operator in both creatively designing the experiment and in its execution. However, none of the techniques described here should be beyond the level of a typical NMR spectroscopist and all can make a tremendous impact on a drug discovery campaign.

## NMR as a tool for quality control

### Solubility and integrity of compounds

There are two vital pieces of information that are absolutely needed for each and every compound assayed in a small molecule drug discovery program: (1) that the compound in the tube is what is expected to be there and is intact and (2) that the compound is soluble at the concentration at which one needs to assay its activity. Both pieces of information are required to properly interpret both biochemical and biophysical assays, as well as to be able to formulate a so-called structure–activity relationship for hit evolution studies (as shown for the simple peptide bacitracin (Epperson and Ming 2000)).

A simple, 1D ^1^H NMR spectrum provides a quick and non-destructive tool to guarantee compound integrity and monodispersity (LaPlante 2013). This includes indications for preferred stereochemical and spatial orientations (diastereomers, cis/trans-isomers, etc.) and tautomeric equilibria (Claramunt, et al. 2006), all of which can influence the binding affinity for a protein (Brink and Exner 2009). Even the most cursory inspection of the spectrum can be used as a quick check that the expected number of resonances are present. Often the ^1^H spectrum of a compound is acquired in an organic solvent upon completion of synthesis. However, we strongly recommend acquiring the spectrum again in an aqueous buffer just prior to using the compound in assays, whether it has been sourced internally or externally. This extra step will ensure that samples have not been accidentally switched or degraded during transport and that the compound is stable and in the desired protonation state in the desired assay (Wan and Ulander 2006).

**Fig. 1.**
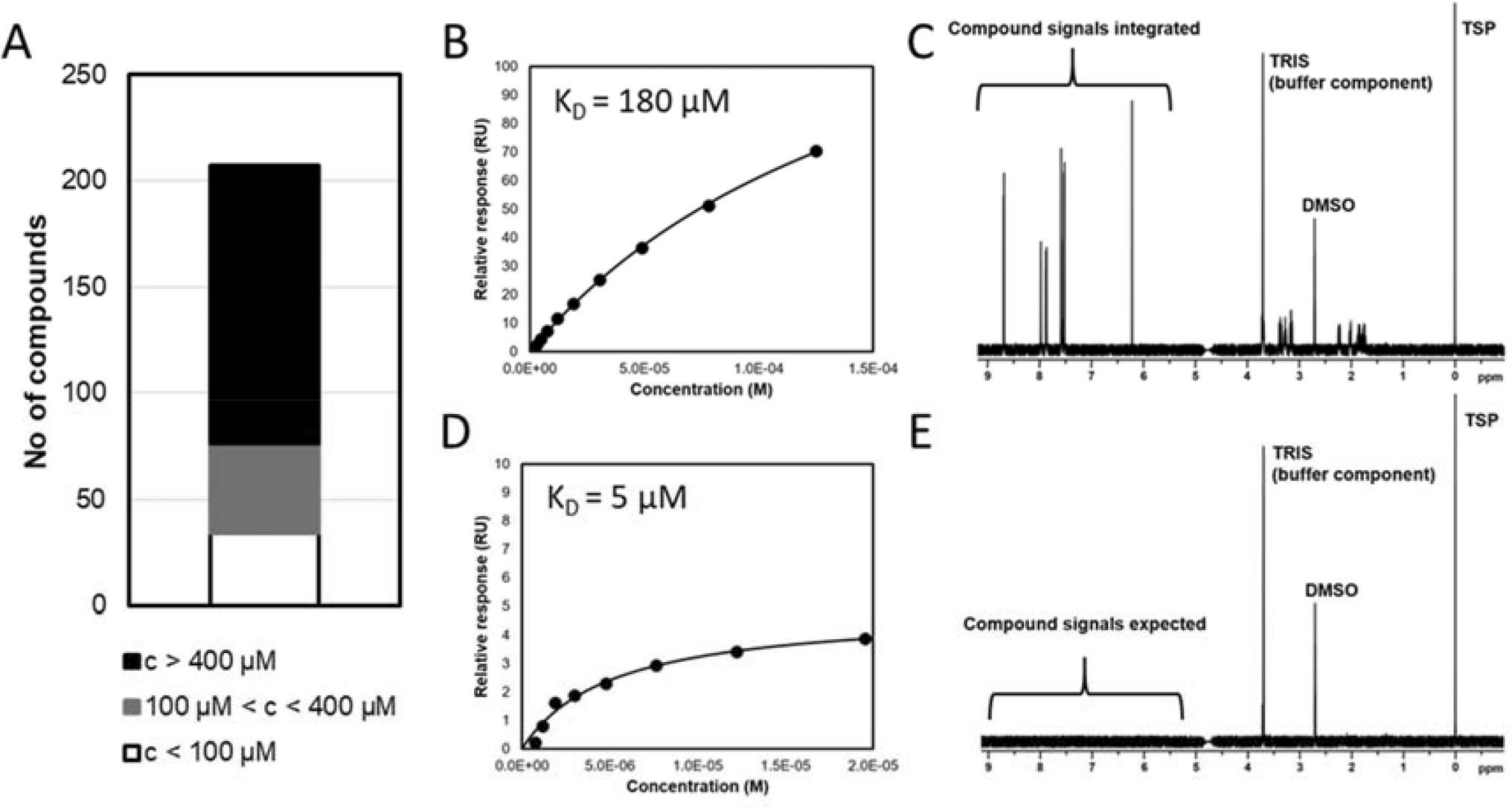
1D NMR assay to assess compound solubility aids in interpretation of SPR data. The solubility profile of fragment analogues in the same chemical class can vary substantially (**A**). The histogram shows the number of compounds in one chemotype that are soluble in one of the three concentration (**c**) ranges. **B** Some analogues exhibit binding curves from SPR that suggest saturation if assayed at higher concentrations. **C** In this case, knowledge that the compound is soluble to significantly higher concentration (here to 500 μM) suggests that the observed data is not an artifact and that the titration could be repeated at higher concentration. **D** In contrast, some analogues exhibit what appears to be ideal Langmuir binding behavior. **E** However, the solubility measurement demonstrates the poor solubility in aqueous buffer. In these cases, the apparent saturation of the SPR curve is likely reporting on the limited solubility of the fragment in solution and the fitted K_*D*_ should be disregarded

In the field of fragment-based drug discovery (FBDD), where potencies are generally low and high compound concentrations are needed for primary and secondary screens, a quick and reliable tool to measure the solubility of a compound is not only useful, but necessary to avoid false positives (e.g. in single injections in surface plasmon resonance (SPR) experiments) or technical ramifications (e.g. disturbance of the microfluidics due to compound aggregation). Any well curated fragment library will have undergone this type of QC analysis. However, during fragment evolution, the properties of analogs quickly diverge from the parent compound while the potency may remain rather low (Kuntz 1999; Leung 2012). As a result, solubility can become limiting for such compounds and result in false positives in a variety of assays typically used in drug discovery cascades that may be difficult to detect and troubleshoot (see Fig. 1).

We have developed a simple variant of the well-known quantitative NMR (qNMR, (Diehl 2020)) method that allows us to rapidly determine the approximate solubility (± 10%). This is done by comparing peak integrals of aromatic protons to that of an internal reference of a known concentration (100 µM trimethylsilylpropanoate, TSP). This is performed in an automated fashion by home written scripts that obtain the integral of primarily aromatic ^1^H’s of the compound and compare this to the integral of the TSP resonance. Typically, we assay at the highest intended concentration of the compound and where the compound is less soluble, we adjust the protocol of the subsequent biophysical assay, e.g. SPR.

Given the low affinity of fragment hits and their close analogs, often it is not possible to titrate to a concentration that is 10× the K_D_ or IC_50_. As a result, it can be difficult to differentiate between a weakly binding, but ideally behaved ligand and one that is not. Figure 1 presents an example of two different analogs of a fragment hit titrated in different experiments. A *prima facie* analysis would suggest that the compound in panel D is more potent than that in B. However, the solubility data strongly suggest that D is artificially solubility limited, consistent with the lower RU response. In contrast, the high solubility of the compound in B suggests that the observed binding curve is artifact free and a reliable measure of the affinity.

In addition to solubility, a number of other compound related artifacts can cause problems with biophysical and biochemical assays. We direct the reader to excellent reviews covering approaches to detect problematic compounds (Zega 2017; Davis and Erlanson 2013).

### Protein

In addition to well characterized compounds, one needs a well-behaved sample of protein as a starting point for a target directed drug discovery campaign. A first critical step in achieving this is of course proper purification, during which we find the use of SEC-MALS (size exclusion chromatography coupled to multi-angle light scattering) to be of tremendous value in ensuring a high level of protein monodispersity (Folta-Stogniew 2006; Wang and Lucey 2003). Subsequently, 1D ^1^H-NMR can also be used to assess the integrity (correct fold and stability) of the protein itself (Dobson et al. 1984). Time dependent characteristics of the protein, such as denaturation or aggregation, can be readily observed (Price et al. 1999; Carver and Lindner 1998). This knowledge allows one to decide whether to screen for buffer conditions that prevent aggregation or design experiments such as biochemical assays more robustly with this in mind. Often the binding of tool compounds with appropriate affinity range (exchange between the bound and free state should be fast on the NMR timescale (Furukawa 2016)) can also be observed at this stage (Stockman and Dalvit 2002; Wang et al. 2004), further confirming the functionality of the protein. Lastly, the effects of various buffer additives, particularly co-solvents such as DMSO (see below), methanol or others on the integrity of the protein can be readily determined, enabling confidence in subsequent assay results.

**Fig. 2:**
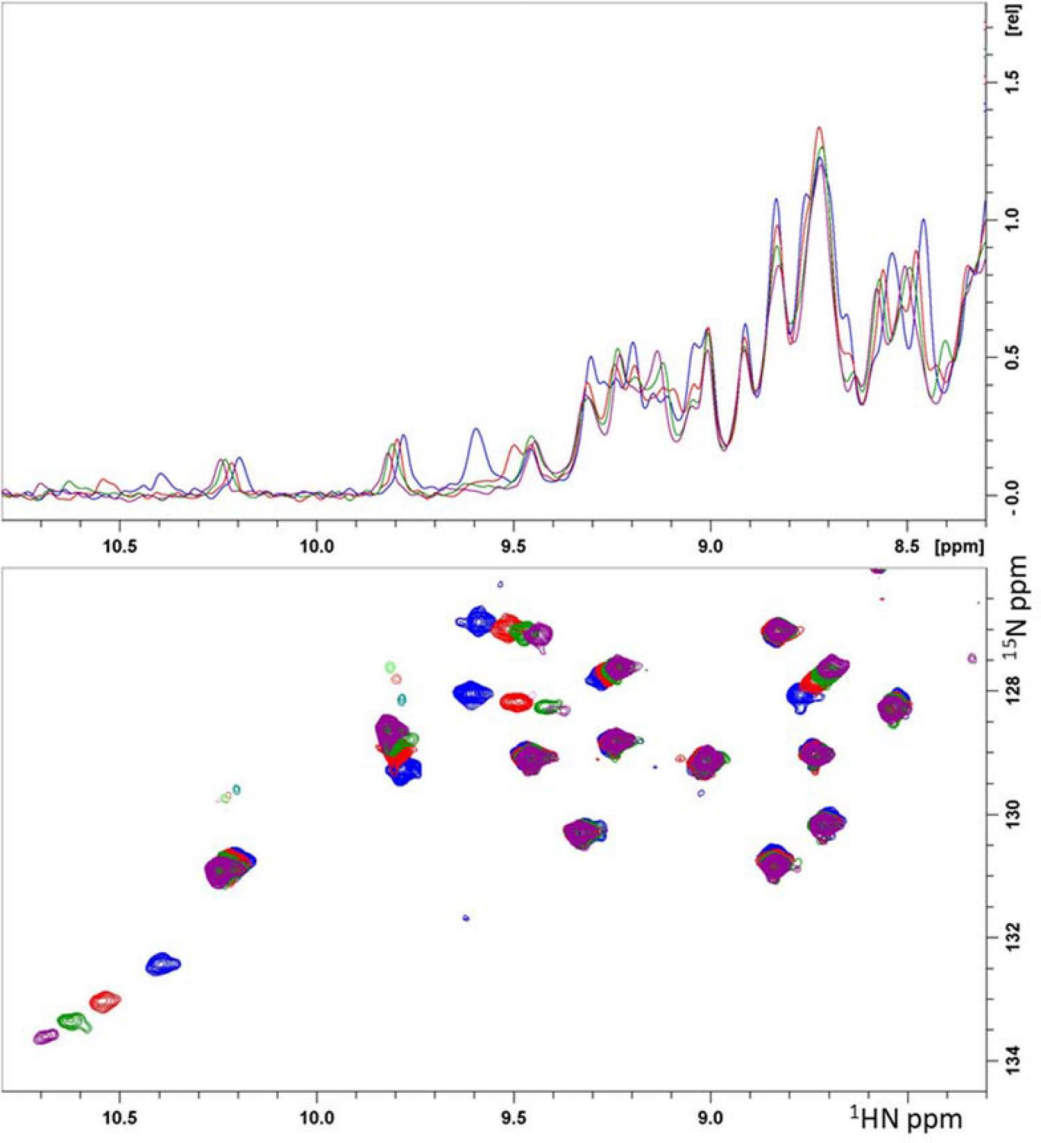
Detection of DMSO binding to a protein target at 100 μM using protein observed NMR spectroscopy. Blue 0%, Red 2% (240 mM), Green 4% (480 mM) and Purple 6% (840 mM) DMSO. Below, a portion of the 2D [^15^N,^1^H] HSQC experiment showing resonances from the protein backbone and the Trp indole resonance. Above, the corresponding 1D ^1^H spectrum indicating the same chemical shift perturbations caused by DMSO binding to the protein

Where isotopic labeling of the target is possible, it enables a far greater level of detailed characterization of protein behavior (Roberts 2000). One underappreciated, yet highly relevant, aspect is the binding of buffer components to the target. In particular, we have noted DMSO binding to a significant percentage of the targets we have encountered (Heightman 2019). Unfortunately, the DMSO often binds to the pocket on the protein that we wish to target (Tjernberg 2006). For ligand finding efforts using High Throughput Screening (HTS), where larger compounds bind with low or sub μM affinity, the presence of a low affinity competitor, such as DMSO, may not be problematic. However, in FBDD, where the affinity of hits is often in the mid μM or even mM range, DMSO at 1–3% (140–420 mM) may be an effective competitor. Moreover, attempts to soak such weakly binding ligands into protein crystals may be thwarted by the presence of DMSO. Fortunately, as shown in Fig. 2, DMSO binding can be readily detected in 2D [^15^N,^1^H] correlation spectra of the protein backbone. In fortuitous cases, isolated peaks in the 1D spectrum may also respond to the binding of DMSO.

It is widely understood that visual inspection of 2D [^15^N,^1^H] correlation spectra, which primarily contain backbone resonances, provides a reliable readout of the solution characteristics of a protein (Clore 1988). This includes the ability to discriminate between folded/unfolded and monodisperse/aggregated proteins (Dyson and Wright 2005). In addition, a slightly more in depth analysis, consisting of peak counting and linewidth determination, can provide insight into whether the target protein is rigid or in dynamic exchange between multiple conformations (Ishima and Torchia 2000). Where desired, this analysis can be extended with partial resonance assignment to infer which portions of the protein exhibit exchange. This information can be of great use if obtaining crystals is difficult, as conformational exchange often prevents crystal formation. Likewise, backbone correlation spectra allow the investigator to discriminate between different possible conformations of a protein (Rabbani 2018). Often the goal is to target one particular conformation of a protein. The allosteric Abl kinase inhibitor developed by Novartis is an elegant example of the use of 2D [^15^N,^1^H] correlation spectra as a conformational readout of the activity/selectivity of a compound (Skora 2013).

### Immobilization

In drug discovery campaigns that make use of biophysical techniques to characterize protein-small molecule interactions, it is often necessary to immobilize the target protein. Our core technologies, one NMR-based and the other SPR (surface plasmon resonance), both require immobilized protein. Immobilization raises the question of whether the protein retains functionality. Although by judicious choice of immobilization strategy we find that in nearly all cases functionality is retained, it is nonetheless imperative to empirically demonstrate this. Where the target is an enzyme, demonstration of activity upon immobilization is the ideal scenario. In many cases it may prove possible to observe enzymatic activity using NMR.

**Fig. 3:**
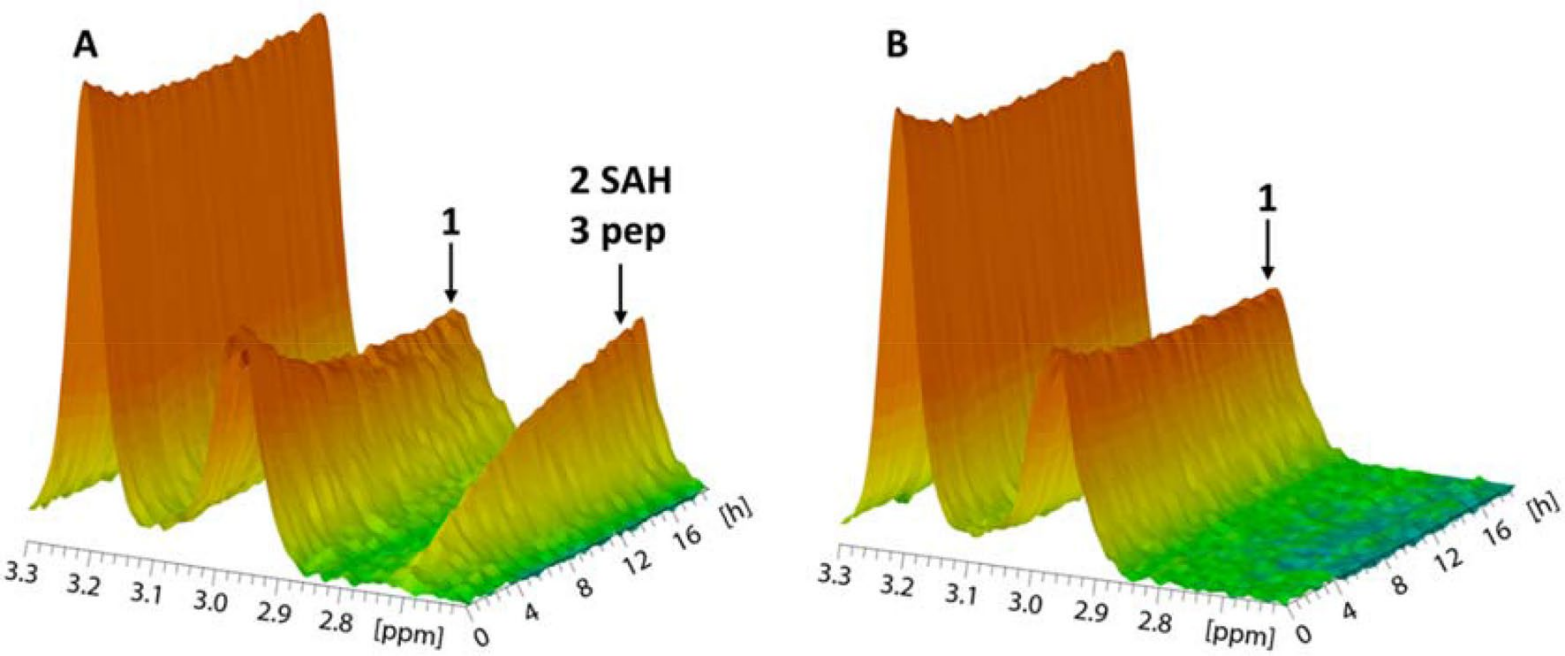
Observation of enzymatic activity through time dependent changes in NMR spectra. 3D overview of the spectral changes occurring during the methyl transfer from SAM to a substrate peptide. Panel A—50 μM immobilized NSD2 and panel B—50 μM immobilized reference protein. Both samples contained 300 μM of a Lysine containing substrate peptide. A portion of the ^1^H spectrum is shown with acquisition once every 10 min for 16 h. In A, a decrease in the intensity of the CH_3_ group of SAM at 2.96 ppm (arrow, 1) is clearly visible. Simultaneously there is an increase in the intensity of two overlapping peaks at 2.7 ppm. One is derived from the methine protons of the homocysteine moiety of SAH (2) and the other from the methylated lysine sidechain of the substrate peptide (3). The concomitant changes in these resonances clearly indicate enzymatic activity of NSD2. In panel B, the slight decrease in the intensity of peak 1 is derived from the spontaneous hydrolysis of SAM. The lack of corresponding peaks 2 and 3 strongly suggest that this is a non-enzymatic process

For one client driven project we were tasked with finding fragment hit matter that selectively bound the methyl transferase domain of NSD2. NSD2 was immobilized via biotin-streptavidin capture and a reference protein, the PH domain of human Akt1, (Hajduk et al. 2005) was similarly immobilized. In TINS (Target Immobilized NMR Screening), the sample of the target and reference protein are in adjacent cells in the probe and we acquire spatially selective ^1^H NMR spectra of each cell (Vanwetswinkel 2005). Figure 3 shows a series of ^1^H spectra of each cell separately acquired over 16 h. A clear, time-dependent loss of intensity of the peak at about 2.9 ppm (peak 1, the methyl resonance of S-adenosyl methione, SAM, the co-factor for methyl transferases) is only observed in the presence of the enzyme, where there is a concomitant rise in the intensity of the resonances at 2.7 ppm (peaks 2 and 3). The peak at 2.7 ppm is the resonance of the methylated lysine of the substrate peptide of NSD2. In the cell containing the reference protein, only the slow spontaneous hydrolysis of SAM can be observed. This assay allowed quantitation of the methyl transferase activity of the immobilized sample during the screening procedure. We could therefore show that the immobilized enzyme was approximately 90% active after the 4.5 days required to screen a library of about 1,500 compounds.

## Fragment screening

Ligand observed NMR methods remain at or near the top of the list of most popular techniques for screening fragment libraries^1^ (Erlanson 2018). There is a substantial body of literature reviewing NMR as a fragment screening tool so we would direct the reader to see any of these (or other) in-depth reviews (Carneiro 2017; Homans 2004; Nitsche and Otting 2018; Sugiki 2018; Gossert and Jahnke 2016; Harner 2017; Dias and Ciulli 2014; Ma 2016). What is appreciated far less widely is the power of ligand observed NMR to begin screening de novo, that is, without any “tool compounds” (small molecules with orthogonally validated binding to the target in a relevant affinity range). Using any of the well-described solution methods, it simply suffices to determine the biological activity of the usually recombinantly produced target and that it is not aggregated under the desired experimental conditions and one is ready to begin screening. SPR, in contrast, absolutely requires a tool compound in order to implement the assay (Perspicace 2009). As a result, we often begin ligand discovery campaigns on unprecedented targets by screening our fragment library using ligand observed NMR. We then select a number of primary hits from the screen and assess the binding in SPR. In all cases, at least one (and usually at least 50%) of the primary NMR hits exhibits binding in the SPR assay. Although the sensorgrams of such initial scouting experiments are typically not ideal, these NMR fragment hits can subsequently be used to optimize assay conditions for fragment screening or HTS hit triage efforts. Using this simple, sequential approach we have successfully carried out fragment screening campaigns against more than 10 unprecedented targets.

## Structural biology

**Fig. 4:**
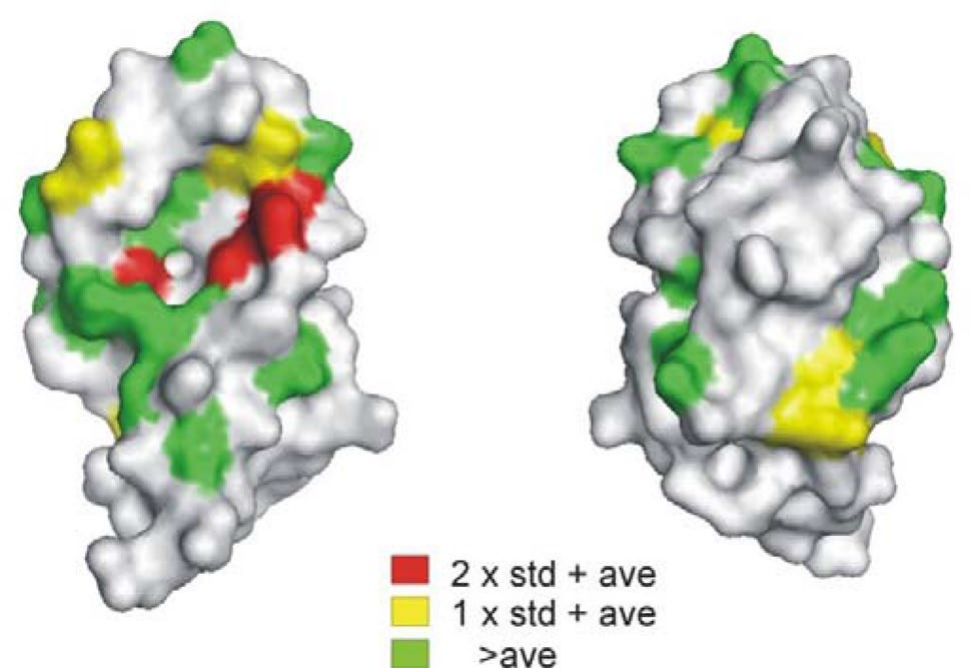
Chemical shift perturbation analysis of a small molecule binding to ^15^N labeled N-terminal domain of hMDM4. Hits from a ligand observed fragment screen were assayed at four concentrations vs control samples containing equal concentration of DMSO. In the example above, the magnitude of compound specific CSPs (The equation that we use for calculating the magnitude of the CSP from heteronuclear data is: ∆(^1^H/^15^N) = (((δ_H_^2^) + (c_N_ * δ_N_)^2^/2)^1/2^ Where c_N_ is a scaling factor to account for the difference in maximally observed CSP for each nucleus. See [48]) was plotted against the amino acid sequence and the standard deviation was calculated. The magnitude of CSPs with respect to the standard deviation has been color coded onto the residue from which it derives. The “hot spot” for fragment binding is clearly formed by the pocket that accommodates the tryptophan side chain of p53. In addition, given the 5 point titration, the solution affinity of the interaction can be estimated. Here the K_D_ was approximately 1 mM

**Fig. 5:**
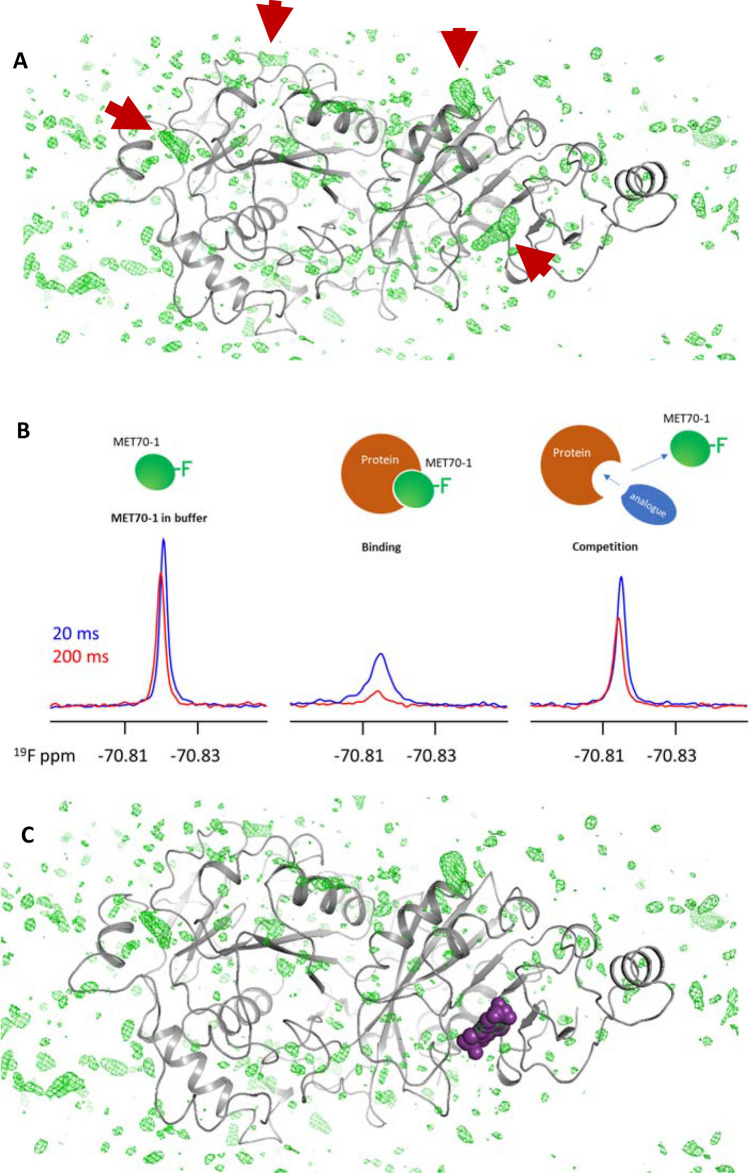
Synergistic relationship between NMR and X-ray crystallography. **A** The ribbon structure of Mettl3/MettL14 is shown placed amongst the residual electron density in the crystal of a complex of a weakly binding fragment hit. Four regions of electron density (arrows) can be identified that in principle have the correct size and shape to accommodate the known structure of the fragment. **B** Competition binding NMR assay to identify the binding site of a ligand. A fragment (MET70-1) containing a C^19^F_3_ moiety was orthogonally confirmed as a hit. On the left, the ^19^F spectrum of the ligand at 2 different T2 delays is shown. Addition of the target results in significant peak broadening, indicating binding of MET70-1 to Mettl3/MettL14 (middle). On the right, addition of an analog, initially SAH, results in peak narrowing suggesting that MET70-1 and SAH compete for binding at the same site. Note the small shifts in resonance frequency are due to sample heating at longer T2 times and protein buffer components. **C** Knowing that the fragment hit was likely bound in the active site allowed confident, unbiased refinement of the diffraction data resulting in the structure shown. The structure could then be used to guide medicinal chemistry efforts

The first and most obvious use of NMR is the well-known binding site mapping using chemical shift perturbations (CSPs), also referred to as SAR by NMR (Shuker 1996). If the backbone resonance assignments of the target are available, then a simple two point titration can be used to identify amino acid residues that respond to the binding of a ligand.^2^ However, this list may contain residues that form the binding site as well as residues remote from the binding site that may be perturbed by a conformational change or changes in the dynamic behavior of the protein (Dehner 2003). Mapping of the CSPs onto the 3D structure often, but not always, reveals a cluster centered around a potential binding pocket (Bonvin et al. 2005). An example is presented in Fig. 4 where the magnitude of the CSP is color coded onto the 3D structure of the N-terminal domain of human MDM4 protein. The cluster of larger CSPs around the pocket that binds the tryptophan sidechain of p53 strongly suggests the site where this fragment hit binds. When the binding site cannot be determined from visual inspection, we find that adding the CSPs of multiple different ligands increases the “signal to noise” as the direction of remote CSPs is significantly more variable than those at the ligand binding site. One caveat is that the various ligands should bind to the same site as determined by e.g. competition binding experiments (see Fig. 5). It is important to note that careful controls are vital to ensure against the presence of artifactual CSPs (Fig. 2). Using this approach, the binding site of up to 20 compounds can be mapped in a weekend. In the early stages of fragment evolution, this is often sufficient information to efficiently guide analoging exercises and at the same time extremely cost effective.

### Synergy between NMR and Crystallography

While some view NMR as competitive with crystallography for providing structural information, in fact the two techniques can be used synergistically. One simple example derives from our efforts to develop inhibitors of the RNA methyl transferase activity of the heterodimeric Mettl3/Mettl14 complex. The construct used for crystallography totaled roughly 85 kDa. Initial electron density maps of the protein-small molecule complex with resolution of about 1.8 Å were available. However, after masking of electron density derived from the protein, a considerable amount remained (see Fig. 5). In principle, this density could be ascribed to buffer/crystallization reagents or to the fragment hit. Since the fragment hit bound weakly (K_D_ ~ 400 μM), it was also possible or even likely that the occupancy within the crystal was less than 100%. Given this situation, it was difficult to assign electron density to the fragment with confidence. Therefore, we sought orthogonal structural information that could aid this process. We used simple ligand observed, competition binding experiments with the cofactor SAM to obtain this information. A number of the confirmed fragment hits proved to be SAM competitive and allowed us to confidently define their binding site within electron density at the cofactor site. Subsequently, we found a fragment analog containing a C^19^F_3_ group that bound at the SAM site. Since this still relatively weak binder was in fast exchange on the NMR timescale, it served as a reporter for the tighter, slowly exchanging compounds that were eventually evolved, using very simple 1D ^19^F spectroscopy, similarly to methods that have been described (Castro and Ciulli 2019).

### NMR vs crystallography

**Fig. 6:**
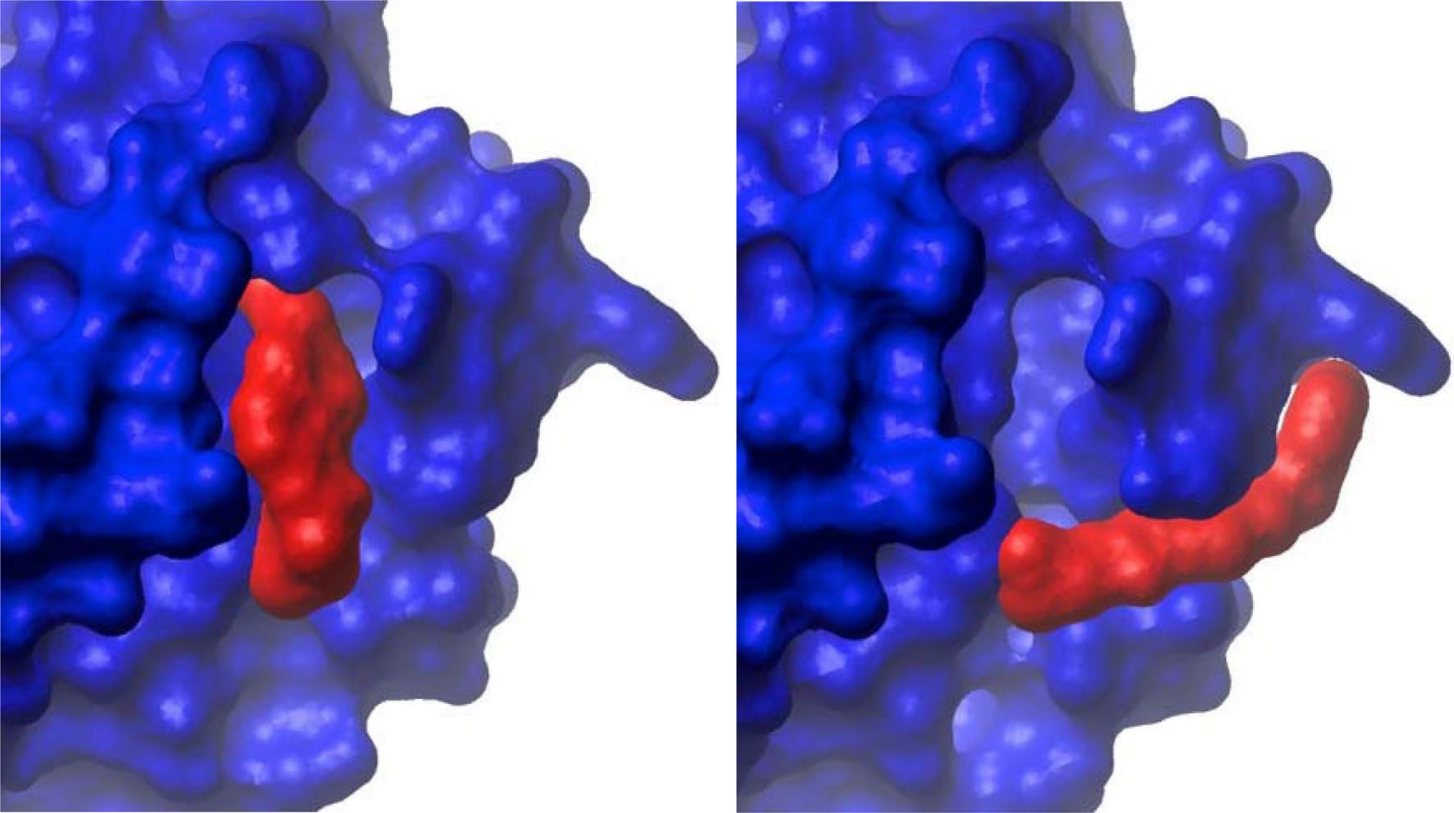
Differences between solution and crystalline state structures. The blue surface is that of a protein and the red a covalent ligand that is bound to it. The structure on the right is derived from high resolution X-ray diffraction data and that on the left from solution NMR. Although the covalent warhead is bound to the same residue, the rest of the molecule occupies a very different position on the surface of the protein. In the crystal structure, the molecule lies in a shallow groove along the surface. A second set of interactions occur with a protein within the adjacent unit cell. In the solution structure, the primary binding pose places the molecule within a deeper groove adjacent to the modified residue. A second, smaller and weaker set of NOEs is consistent with the crystal structure suggesting that in solution, the molecule exchanges between two binding poses with the major one shown on the left.

One obvious difference often noted between crystallography and solution NMR is the state of the sample. While in the majority of cases, structures solved independently using the two methods are (nearly) identical, this is clearly not always true. We have encountered numerous cases where the so-called structure–activity-relationship data (that is some measure of potency compared to small changes in the structure of the compound) was not explained by the crystallographic data. Often this is due to an artifactual binding pocket created at the interface between two protein monomers within the crystal lattice. Since such a pocket would not exist in solution, when technically feasible, NMR can provide the more relevant structure. *A priori* one might expect such artifacts to appear more frequently with weakly bound ligands such as fragment hits. However, this too is not always the case. Figure 6 illustrates an example of an irreversible covalent ligand that can nonetheless occupy two rather different grooves on the surface of a protein. In solution (left panel) NOEs consistent with two poses are found. In the major pose, the non-covalent contacts with the protein lie in a deep groove on the protein surface. A second set of weaker NOEs were also observed. Within the crystal lattice the compound exhibits a unique binding mode occupying a shallow groove on the protein surface. However, additional contacts are made to an adjacent protein molecule in a different unit cell. The crystal structure is consistent with the weaker NOEs suggesting that in solution, the ligand exchanges between the two binding poses. Apparently the extra contacts from the lattice increase the affinity of the second pose sufficiently that it dominates in the crystalline state.

#### Summary

NMR is a powerful, information rich tool that can provide critical information at many stages of the pre-clinical drug discovery process. Here we have only presented examples from the very early stages of target preparation and hit discovery. Even here however, there exist multitudes of examples where NMR has provided key information that has enabled drug discovery programs against challenging targets. Beyond this early stage, the non-destructive nature of NMR offers the potential to observe protein–ligand interactions in more complex environments. A recent review discusses examples of how NMR can be used to address target engagement questions within living cells (Siegal and Selenko 2019). While work within this area is not yet ready for industrial scale implementation, the exciting discoveries being made will surely make significant contributions to our understanding of how drugs behave in cells and perhaps even in organs in due time.

## Footnotes

1 https://practicalfragments.blogspot.com/2019/12/poll-results-affiliation-and-fragment.html.

2 If assignments are not available, we find that the combination of Non-uniform sampling (NUS) with automated assignment procedures such as FLYA (Schmidt and Güntert 2012) is able to yield sufficient assignments to map binding sites in 4–6 weeks for proteins up to about 40 kDa, assuming appropriate isotope labeling schemes are practical.
